# Clinical Evaluation and Treatment of Patients with Postconcussion Syndrome

**DOI:** 10.1155/2021/5567695

**Published:** 2021-05-29

**Authors:** Vijay Renga

**Affiliations:** Dartmouth-Hitchcock Medical Center, Geisel School of Medicine at Dartmouth, Lebanon, NH 03756, USA

## Abstract

Postconcussion syndrome (PCS) is a complex set of symptoms occurring in a small percentage of patients following concussion. The condition is characterized by headaches, dizziness, cognitive difficulties, somatosensory issues, and a variety of other symptoms with varying durations. There is a lack of objective markers and standard treatment protocols. With the complexity created by premorbid conditions, psychosomatic issues, secondary gains, and litigations, providers often find themselves in a tough situation in the care of these patients. This article combines literature review and clinical insights with a focus on the underlying pathophysiology of PCS to provide a roadmap for evaluating and treating this condition.

## 1. Introduction

Concussion is a common injury in all age groups. While most patients recover from the impact with no long-term sequelae, a small proportion go on to develop a constellation of symptoms known as postconcussion syndrome (PCS). These symptoms are mostly subjective and include mood changes, fatigue, memory problems, dizziness, headaches, and diffuse pains. Occasionally, clinical signs such as eye movement disorders and neck muscle dysfunction can be elicited.

The type and severity of initial injury may have no bearing on the occurrence or severity of PCS. Management of PCS is challenging because of the paucity of clinical, radiographic, and electrophysiological findings. The entire assessment and treatment often depends on the patient's subjective symptoms. Add to this, the complexity created by worsening of preexisting psychosomatic disorders, substance use issues, medication side effects, litigations, secondary gains, and compensation neurosis, the entire picture can get muddied. The involvement of different specialties such as occupational medicine, ENT, ophthalmology, rehabilitation, neurology, neurosurgery, neuropsychology, and psychiatry can further complicate the situation.

With limited objective measures of disability, wide-range of symptoms, and no proven treatment protocols, providers often face a tough situation in the care of these patients. The aim of this article is to improve the knowledge of PCS, elucidate the underlying pathophysiologic basis, and provide a roadmap for evaluation and treatment of this condition. We will also look into newer and upcoming imaging and electrophysiologic modalities that can help us objectively assess PCS.

## 2. Methodology

This article combines literature review with clinical insights into evaluation of postconcussion syndrome. Literature search was carried out using Medline with search terms mild TBI (mTBI) and postconcussion syndrome. Articles pertaining to etiology, pathophysiology, and management that are relevant to clinical practice were selected for this manuscript.

## 3. Epidemiology

Head injuries are very common whether it be from sports, motor vehicle accidents, workplace injuries, or falls. 

The course begins with patients being brought to the emergency department (ED) from the incident site with a head or neck impact. In the ED, they often undergo a CT scan of the head and sometimes of the cervical spine, which are almost always normal. They get discharged home with the advice to “take it easy” for a few days and follow-up with their primary care physicians (PCP). Other patients who do not visit ED initially, approach their PCP or an urgent care in a few days of the injury complaining of headaches, dizziness, or fatigue. In either scenario, a diagnosis of “concussion” is often made.

Concussion is defined as a mild traumatic injury to the brain, causing physiological disruption in brain activities with no apparent structural damage on a CT scan or MRI brain. Immediate symptoms are transient loss of consciousness lasting less than 30 minutes with a Glasgow Coma Scale of 13–15 with or without amnesia.

Headaches are common during the acute phase of concussion. Transient amnesia may also occur. Rarely convulsions are seen. The prevalence of concussion is difficult to estimate, since it is very common and often under reported. MildTBI (mTBI) occurs more in the young and the old with a male predominance. In young patients, there is often full recovery by 2 weeks and three-fourths of patients recover by a month, and almost 90% recover by 2 months [1].

### 3.1. Postconcussion Syndrome

While the majority of concussion symptoms dissipate, a constellation of symptoms can persist or worsen including headaches, dizziness, blurry vision, difficulty with concentration and processing, memory changes, problems with multitasking, mood issues with irritability, and sensitivity to light and sound. These symptoms are grouped into postconcussion syndrome. Ten to 30 percent of patients with a mild TBI (mTBI) go on to develop postconcussion syndrome [2]. Most symptoms except headache and neck pain are not usually present at the time of injury and develop over a few days to a week. Patients who were discharged from the ED or seen by PCP return to them with a cluster of these symptoms. They can often be unhappy with their providers for not discussing the possibility of postconcussion syndrome and not advising enough rest at the time of initial injury. On the other hand, for the ED providers or PCPs, it is often difficult to predict which of these head injury patients were likely to develop PCS [3]. Once the symptoms set in, it can worsen and last from weeks to months with a wide variability in symptomatology, severity, and duration depending on various factors.

Patients who lose consciousness during the head impact tend to have higher incidences of PCS than those who remain conscious. The severity of the injury does dictate the duration of recovery [4], eventhough patients with major TBI and structural damage with subdural hemorrhage or cortical injuries could have lesser incidence of postconcussion syndrome than those with a trivial head impact or a whiplash. No individual factor can be a definite predictor for developing PCS, but the “symptom burden” at the time of injury or the multiplicity of symptoms bears a significant relation to patients developing postconcussion syndrome [1, 5]. These symptoms include headache, nausea, vision problems, dizziness, tinnitus, neck pain, the history of concussions, psychological issues, alcoholism, substance use, and migraines. ED physicians and PCPs should focus on these subsets of patients when discussing the possibility of developing postconcussion syndrome.

### 3.2. Defining Postconcussion Syndrome

Postconcussion syndrome is the persistence of concussion symptoms beyond expected duration for recovery. The ICD-10 diagnostic criterion for postconcussion syndrome (310.2) is as follows [6].

- History of head trauma within 4 weeks associated with three or more of the following 8 symptom categories
- Headache, dizziness, malaise, fatigue, phonophobia
- Irritability, depression, anxiety, emotional lability
- Attention/memory deficits without any neuropsychological evidence of significant impairment
- Sleep difficulties
- Alcohol intolerance
- Psychosomatic symptoms attributed by patients to brain damage and assuming a sick role

## 4. Etiology of Postconcussion Syndrome

Postconcussion syndrome (PCS) is often considered a “functional” disorder due to the paucity of imaging abnormalities. Often, this is not the case as both structural and functional injuries of varying degrees occur that are independent of the type or severity of injury. These changes are inconspicuous on routine imaging techniques using CT scan or MRIs.

**Figure 1:**
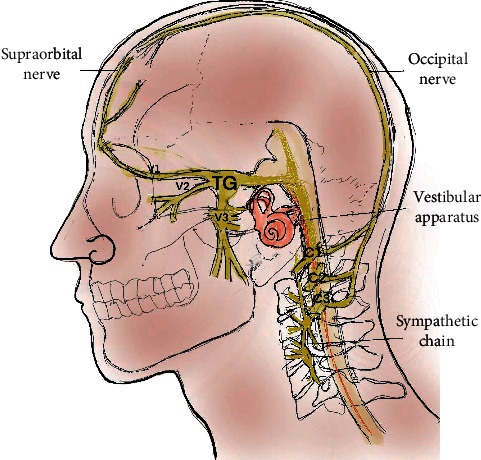
Cervicovestibular trigeminosympathetic system: the anatomic and functional connections between the trigeminal, vestibular, cervical, and sympathetic systems which are often involved in postconcussion syndrome. Cervical roots and the trigeminal system run proximal to each other with convergence of their afferents. The vestibulocollic and vestibulospinal tract controls the neck and truck tone. Cervical roots are in close proximity and communicate with the cervical sympathetic chains which are connections to the autonomic nervous system. TG, trigeminal ganglion; C1/2/3, cervical roots.

In addition to intracranial impacts, there are whiplash injuries to the cervical roots, vestibular system, and neck musculature causing extracranial effects. These extracranial effects lead to headaches, dizziness, vision, and balance problems (Figure 1).

Functional components on the other hand are primarily related to the impact on the brain tissue when it gets shaken up. It causes disturbances in cognition, mood, and discriminatory functions.

The functional and structural components are not mutually exclusive, often intertwined and difficult to parse out. Combined, they form the crux of PCS. Therefore, it is important to understand each of these components separately.

### 4.1. Intracranial Components and Effects

Mild TBI generally impacts the frontal and frontotemporal regions. There is axonal strain and shear in the cortex and brainstem. It causes neuronal membrane disruptions leading to ion fluxes and abnormal depolarization [7]. Shearing may not manifest initially and remain invisible on routine imaging. It can be identified with techniques such as diffusion tensor imaging (DTI) as a change in diffusivity of water molecules along the axonal tracts.

More problematic is the disruption of functional networks of the brain. There are over 100 billion neurons in the brain, which are interconnected by trillions of networks. Functional MRI using blood oxygen level dependent (BOLD) imaging has shown the disruptions of these functional networks following TBI [8]. These changes are not immediate and happen over a few days to a week following injury. Such metabolic and hemodynamic changes can also be identified using positron emission tomography (PET scan) and arterial spin labeling studies (ASL) [9].

Certain neurohistochemical changes are characteristics of more severe TBI. Membrane injury leads to potassium efflux and calcium influx, mediated by the glutamatergic mechanism causing a rise in free radicals and mitochondrial dysfunction [10]. Repeat injuries lead to cell dysfunction and death [7, 11]. There is also a reduction of N-acetyl aspartate (NAA) in injured tissues, which can be assessed using MR spectroscopy. Neurovascular coupling becomes inefficient even with a normal or increased cerebral blood flow (CBF) [12], indicating a functional mismatch [13]. Such flow changes at the macroscopic level can be assessed using transcranial Doppler (TCD) and functional near infrared spectroscopy (fNIRS) [14].

Pain occurs from triggering of the trigeminothalamic system. Trigeminal activation starts from orofacial cutaneous impulses or neck musculature [15, 16]. Whiplash injuries can activate cervicotrigeminal convergence mechanisms [17]. Such convergence reflex can be clinically seen by stimulating the supraorbital nerve (trigeminal branch) while recording the sternocleidomastoid (cervical root). Reciprocally, the stimulation of the greater occipital nerve increases the excitability of dural afferents, triggering the trigeminal system.

The vestibular system maintains the neck and trunk stabilization through vestibulocollic and vestibulospinal reflexes. Brainstem and peripheral vestibular impairment causes deranged neck tone and spinal balance mechanisms. Vestibuloocular reflex involvement can impair visual target stabilization leading to blurry vision.

### 4.2. Extracranial Components and Effects

Neck muscle spasm from cervical root irritation or trauma can impair the reciprocal cervicovestibular and vestibulocervical feedback mechanisms.

The most common and least identified injury from acceleration deceleration injury is a whiplash impact on the cervical musculature and the cervical roots. The cervical musculature surrounding the first 3 vertebrae have high concentration of proprioceptors, which communicate with the inferior vestibular nuclei. They mediate the cervicospinal reflexes, which are important in postural reflexes and balance mechanisms. These reciprocally communicate with the vestibulocollic, vestibulospinal, and vestibuloocular reflexes [18]. This cervicooculovestibular dysfunction is at the core of most PCS-related symptoms [19]. Cervical proprioceptors synchronize with vestibuloocular and optokinetic reflexes to maintain a visual target at fovea during head movements. Tightening of neck muscles from cervical root irritation limits neck ROM and convergence mechanisms resulting in double vision, dizziness, lightheadedness, and imbalance [20, 21].

Central and peripheral autonomic involvement also occurs in PCS. The sympathetic supply to the head and neck originates from the superior middle and inferior cervical ganglion. The superior cervical ganglion lies proximal to the C1 and C2 vertebrae and often involved in head and neck trauma. The stellate ganglion gives branches to the heart and blood vessels. Impact on these can cause dysregulation leading to tachycardia and blood pressure fluctuations. In addition, central autonomic dysregulation also leads to heart rate and blood pressure changes. Ipsilateral partial Horner's syndrome is a common feature of neck injuries. On the other hand, sympathetic overactivity can also occur leading to mydriasis, facial flushing, and increased sweating.

A cervico-oto-ocular reflex proposed by Franz et al. links the trigeminal, vestibular, and oculomotor systems with the cervical sympathetic system. Eustachian tube dysfunction and Meniere's disease can result from impairment of this proposed reflex mechanism [22]. There is communication between the intracranial spaces and inner ear through the perilymph, the internal auditory canal, and the endolymphatic sac [23]. The CSF pressure changes from head trauma can thus affect the vestibular system through pressure shifts within the system.

## 5. Clinical Features of Postconcussion Syndrome

Clinical symptoms whether arising from the intracranial or extracranial causes can be grouped into 5 categories.

(i) Cognitive:
  1. Memory deficits
  2. Attention and concentration difficulty
  3. Difficulties with speech
  4. Executive dysfunction
  5. Fine motor difficulties
(ii) Psychologic
  1. Depression
  2. Anxiety
  3. Irritability and personality changes
  4. Fatigue
  5. Derealization
(iii) Somatosensory and vestibulocochlear dysfunction
  1. Headaches
  2. Nausea and vomiting
  3. Light and sound sensitivity
  4. Hyperalgesia
  5. Tinnitus
(iv) Visual symptoms and oculomotor dysfunction
  1. Light sensitivity
  2. Blurry vision
  3. Convergence difficulty
  4. Double vision
  5. Horner's syndrome
(v) Autonomic symptoms
  1. Heart rate and blood pressure fluctuations
  2. Abnormalities of sweating and pupillary abnormalities
  3. Temperature dysregulation
  4. Sexual dysfunction
  5. Sleep alterations with poor sleep efficiency

### 5.1. Cognitive Deficits

Neuropsychological issues are the most disabling problem in postconcussion syndrome. This results from mechanical impact on the brain, especially the frontal region. The frontal lobe plays a major role in initiation and sustenance of attention, shifting focus, and refocus [24]. Attention, speech, and executive dysfunctions occur in PCS. Loss of attention and concentration contributes to a false perception of memory issues when the core issue in most patients is the lack of attention. Functional disruption affecting the resting brain networks, especially the default mode network (DMN), has been demonstrated in TBI [25].

Frontal lobe dysfunction symptoms include the following:

1. Attention deficits: postconcussion effects can mimic an ADHD-type disorder [26]. Besides difficulty focusing on the task at the hand, multitasking is severely impaired. Cognitive endurance is limited, and patients often notice routine day-to-day tasks becoming cumbersome. This is a frequent symptom that makes patients approach caregivers.
2. Speech problems: functional speech difficulties often occur with postconcussion syndrome. Stuttering is a common feature. Such stuttering peaks during initiation of speech and wanes as the patient gets into a flow. They worsen with stress and attention to the impediment. An isolated stutter is often associated with psychological issues, worker's compensation, or ongoing litigation. They notably improve with distraction.
3. Irritability: patients with PCS are hyperirritable with limited emotional regulation. Spouses and family members take the brunt of their aggression. Emotional lability with crying and bouts of anger are common. Patients can flip out on trivial issues during social interactions.
4. Mood and personality changes: depression and mood issues are integral to PCS. Incessant thoughts about their disabilities often lead to anxiety and depression. PTSD related to inciting events, suicidal ideation, and substance abuse tends to occur in these scenarios.
5. Loss of executive function: planning, organization, and execution of daily tasks are severely affected. Poor performance at work with frequent mistakes and problems with group interactions is common. Anxiety related to underperformance leads to more psychological stress and deterioration of work capacity.

Despite frontal lobe dysfunction, inappropriate and disinhibited behavior as seen in frontotemporal dementia is rare in PCS because of an intact insight. This insight, however, leads to worrying that can be counterproductive to the recovery process. Expecting a poor prognosis often becomes a self-fulfilling prophecy.

### 5.2. Somatosensory Dysfunction

While not as prominent as frontal lobe issues, parietal and somatosensory dysfunctions do occur in PCS. There is poor discriminatory function causing increased sensitivity to light, sound, pain, and other sensory stimuli leading to photophobia, phonophobia, and hyperalgesia. Tinnitus, nausea, and dizziness are common. Hypersensitivity can trigger migraines and myofascial pains. Changes in smell, taste, appetite, gastrointestinal, and sexual dysfunction also occur.

### 5.3. Depression and Anxiety

Mood changes with depression and irritability are very common in postconcussion syndrome. Corticolimbic models of depression have been hypothesized where reciprocal networks of frontolimbic subcortical systems are affected following TBI [27]. FMRI studies show reduced activation in the dorsolateral prefrontal cortex and striatum and reduced deactivation in the medial frontal and temporal regions [28]. Individual factors play an additional role with exacerbation of preexisting mood disorders.

Similar to depression, anxiety is very common with postconcussion syndrome. Many factors contribute to anxiety including intact insight of cognitive deficits, underperformance at work, and fear of social situations. Up to 80% of patients with PCS have shown clinical anxiety, and their anxiety levels are correlated with the severity of postconcussion syndrome at different time intervals following a head injury [29].

### 5.4. Fatigue and Sleep Derangement

Fatigue is one of the earliest indicators of postconcussion syndrome [30]. PCS limits both physical and psychological endurance. Patients get fatigued and are unable to continue working within a few hours of starting their routine jobs. There is often sensory overload in workplaces that could be contributory.

Daytime sleepiness is common. Hypersomnia and insomnia are both prevalent in PCS [31]. Quality of sleep is often poor with delayed sleep onset and nonrefreshing sleep. The sleep quality is poor in all stages of sleep [32]. Sleep apnea and psychosocial factors can become additional confounders that contribute to sleep disruption. From sleep disruptions, fibromyalgia type symptoms emerge with diffuse aches and pains.

### 5.5. Headache

Headache is the most common presenting symptom of postconcussion syndrome. After a mild TBI, 30–90% of patients may have a persistent headache. Brain tissue is “insensitive” to but “responsive” to pain. The receptors for pain are located in the meningeal layers, periosteum, skull, cervical vertebrae, and scalp. Pain pathways ascend to the spinal nucleus of the trigeminal nerve in the upper spinal cord where the cervical afferents converge on to them. This sets up reciprocal circuits where neck problems trigger migraine headaches and vice versa (Figure 1). Impaired discriminatory and modulatory functions cause lowered pain threshold and tolerance leading to easily triggered, unrelenting headaches in PCS. Poor sleep and increased inflammatory neuropeptides also plays a role.

Like PCS, occurrence of chronic posttraumatic headaches can be inversely related to the severity of the TBI [33]. An individual with a major TBI and intracranial bleeding can have a lower risk for chronic headaches compared to somebody with just a whiplash injury. In the young and adolescents, posttraumatic migraine is the most common type of headache [34]. Whiplash injury sets off cervicogenic headaches that spread from occipital regions to the forehead. Retroorbital pain is the characteristic feature of these headaches. Occipital neuralgia-related headaches become chronic and refractory to treatment because of its location in the neck, where it is subject to constant torsion from neck movements. It triggers migraine episodes by reciprocal trigeminal mechanisms. Cervical root irritation leads to neck muscle spasm, which causes further irritation of the cervical roots generating a vicious cycle. Upstream and downstream manifestations occur with upper cervical root irritation triggering headaches and atypical facial pains, while lower roots cause paresthesia and pain of the arm and hands. Rarely, patients may experience paresthesia in the legs from spinal cord irritation. The cycle created by the nerve irritation and the neck muscle spasm feed each other leading to hypersensitivity, unrelenting headaches, dizziness, vertigo, and overall decreased quality of life in these scenarios.

### 5.6. Impaired Sensory Discrimination

As described before, light and sound sensitivity is common following concussion due to hypersensitivity. Hyperalgesia, tinnitus, and myofascial pain symptoms predominate. Such sensory and auditory gating abnormalities have been shown in mTBI patients using paired auditory evoked P50 responses.

### 5.7. Vestibular Dysfunction

Balance depends on the vestibular system, peripheral sensations, and vision. Eventhough the peripheral vestibular system is protected inside the temporal bones, head impacts can often cause transient or permanent dysfunction of the vestibular apparatus. The vestibular system is connected to the cervical musculature via the vestibulocollic reflex, the ocular system with vestibuloocular reflexes, and the spinal musculature and righting reflexes through vestibulospinal tracts. It is also connected to the autonomic nervous system through ill-defined vestibular autonomic pathways. Postconcussion syndromes are often associated with some degrees of vestibular dysfunction, which are from direct involvement of vestibular apparatus or secondary to brainstem and cortical impacts. These reciprocal systems dysfunctions impair each other.

Dizziness or lightheadedness occurs in most cases. Peripheral vestibular involvement is more common than central causes following TBI. Unilateral and peripheral pathologies often lead to vertiginous symptoms, whereas bilateral and central lesions lead to lightheadedness [21].

### 5.8. Derealization

Following concussion, patients often describe a sense of detachment from reality or being in a haze. While derealization can be a psychological symptom, the underlying etiology is likely vestibular from the involvement of the otolith organs. The utricle and saccule play a role in our sense of reality. Utricular stimulation has been shown to cause derealization spells [35].

### 5.9. Visual and Oculomotor Abnormalities after TBI

Most visual and oculomotor symptoms after TBI are explained by vestibular dysfunction, sensory discrimination deficits, and migraines. Eye movement dysfunction is a prominent feature in PCS which are likely from brainstem dysfunction. Vergence disorders, especially convergence insufficiency, are common after mTBI. It interferes with reading, binocular vision, and depth perception [36]. Saccadic dysfunction is also seen which is likely due to cortical dysfunction. Frontal lobe injury is characterized by deficits in antisaccades when an attempt is made to move the eyes against a visual stimulus. Smooth pursuit movement and visual tracking are also impaired due to parietal lobe involvement. Vestibuloocular reflexes become dysfunctional due to reciprocal impairments in vestibular and oculomotor systems.

### 5.10. Autonomic Dysfunction

Autonomic dysfunction is commonly seen following concussion [37]. Both central and peripheral sympathetic dysfunctions may occur. Cardiac conduction changes, dysregulated changes in heart rate variability to forced breathing, and R-R interval changes and BP variability with standing and valsalva have been described after concussion [38]. Central autonomic dysfunction occurs due to perfusion abnormalities, baroreceptor reflex dysfunction, and sympathetic abnormalities [39]. Peripheral sympathetic dysfunction with attenuated cardiovascular responses to cold pressor tests has been described [40]. Cervical sympathetic dysfunction can cause Raynaud's and complex regional pain syndrome (CRPS) in upper extremity [41]).

## 6. Evaluation of Postconcussion Syndrome

Evaluation of PCS is primarily clinical. A proper history can help isolate worsened preexisting conditions from new postconcussion symptoms.

### 6.1. Clinical Examination

There are specific elements to be focused on clinical examination. These include the following.

1. Vestibulocochlear system: the hearing test with a finger rub may reveal a hearing asymmetry that was previously inapparent to the patient. This may indicate a cochlear damage on the affected side, which often coexists with a vestibular dysfunction. Nystagmus with a gaze to the affected side is often seen. Vestibular balance testing with a Sharpened Romberg involves having a patient trying to stand on a line with the eyes closed and hands crossed. There is often imbalance and a sway to the affected side in case of cervical or vestibular dysfunction. Dix–Hallpike maneuver of the affected side often precipitates symptoms of dizziness and nausea and occasionally nystagmus.
2. Autonomic dysfunction: pupillary asymmetry can occur from sympathetic dysfunction, resulting in a partial Horner syndrome with smaller pupils on the affected side with or without ptosis which indicates a sympathetic dysfunction. Cold and sweaty extremities, dilated pupils, orthostatic hypotension, and tachycardia indicate sympathetic dysfunction
3. Brainstem and cortical function: extraocular movements with saccadic and smooth pursuit movements elicit nystagmus and convergence deficits. Isolated nerve palsies, especially of the trochlear nerve, can be seen.
4. Neck dysfunction: paraspinal neck muscle tenderness and spasm, occipital neuralgia, limited neck ROM, positive Spurling's test, and brisk deep tendon reflexes are all indicative of cervical involvement. Dizziness on vestibular shake up testing may give clues to underlying cervical and vestibular dysfunction.
5. Cognitive function: A MOCA testing (Montreal Cognitive Neurologic Assessment) can provide a quick and useful screening of cognitive, visuospatial, executive, attention, and language functions. Comprehensive evaluation with neuropsychological testing can be ordered based on the results of this.

A Rivermead Postconcussion Syndrome Questionnaire is the most used evaluation tool specific to PCS [42] that can help track symptomatology over a long time.
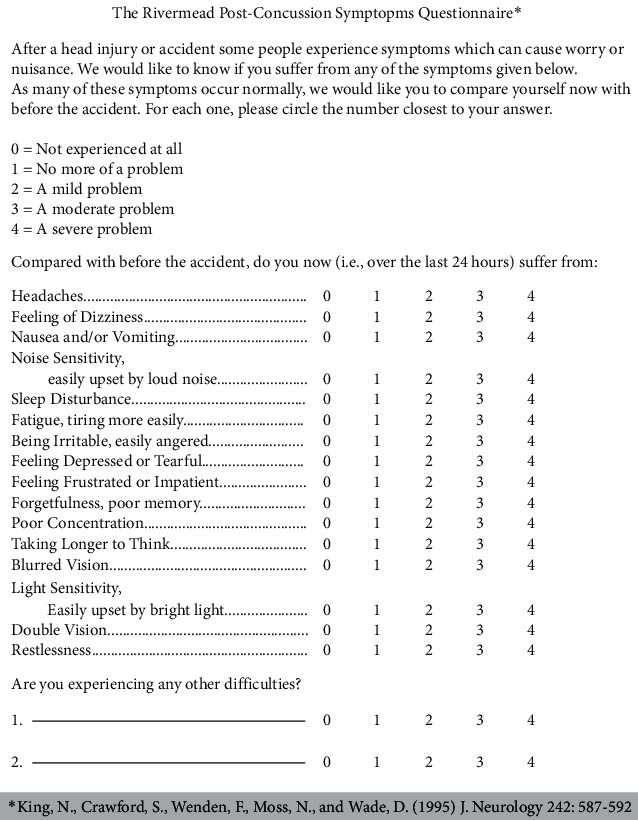


Routine imaging and electrophysiologic studies with CT head, MRI brain, and EEG are often unyielding from a diagnostic perspective, but can reassure the patient that no structural damage occurred. X-rays of the neck can be considered for spine issues. They rarely show any evidence of injury from mild TBI or whiplash injury but can identify contributory issues with cervical spondylosis. Cervical spine MRI is more useful for this, but not routinely performed since preexisting and incidental cervical disc diseases can confound the clinical picture. If there are myelopathic features or active radiculopathy symptoms with severe shooting pains and extremity weakness, then MRI is indicated. Susceptibility weighted sequences on MRI brain may show evidence of hemosiderin deposition in regions of old injury.

Rarely, mTBI can be associated with seizures in the hyperacute phase. Posttraumatic epilepsy can occur in more severe cases. Electroencephalogram (EEG) has low sensitivity for mTBI but occasionally shows focal or diffuse slowing, which may not be of much diagnostic value. The chances of detecting abnormalities on EEG are higher immediately following a head injury, but getting an EEG is not feasible in most acute trauma situations. Clinical and electrophysiologic correlation of EEG is poor for PCS. Magnetoencephalogram (MEG) and quantitative EEG may have greater sensitivity in detecting abnormalities. MEG changes were seen in up to 10% of patients after mild TBI. The same proportion of people go on to develop postconcussion syndrome, but it is not clear if MEG abnormalities have any predictive value for PCS.

Functional imaging is more useful than structural images for mild TBI. SPECT scans show reduced regional cerebral blood flow in mTBI. However, spurious findings are common. A normal SPECT scan in mTBI settings has more prognostic value than an abnormal scan [43, 44]. PET scan has shown frontal or anterior frontotemporal hypometabolism in patients with mild TBI with persistent neurocognitive deficits and normal MRI or CT scans [45]. Structural imaging using diffusion tensor imaging (DTI) in postconcussion syndrome following mild-to-moderate TBI has shown reduced fractional anisotropy (FA) and mean diffusivity (MD) along the white matter tracts. The diffusion parameters in several regions correlated with the assessment scores in postconcussion patients [46].

Quantitative EEG and fMRI are promising technologies in postconcussion syndrome evaluation [47]. QEEG measures the frequencies using spectral analysis and coherence analysis. It measures the frequency synchronicity between different scalp regions, which indicates network connectivity [48]. Findings in mTBI included reduction of the mean alpha range frequency and increased theta and delta range activity [49], with an increased alpha-theta ratio. Statistical analysis of QEEG has developed discriminant functions that can distinguish between mTBI and controls. Spectral analysis and coherence measures of EEG were used to create the variables for discriminant functions with 80–95% discriminant accuracy [50].

Functional MRI with cognitive tasks has shown differing activation patterns for cognitive tasks in controls compared to mTBI patients [51]. Hype-activation of brain regions for similar tasks was seen in PCS patients [52]. These changes resolve with resolution of symptoms, spontaneously or with rehabilitation. At the same time, there were underactive regions of the brain in PCS which could be regions of structural damage or dysfunction secondary to the impact. Increased activation was seen in subcortical regions besides cortical regions in PCS patients. On the other hand, connectivity studies using resting state fMRI studies in mild TBI patients at separate intervals showed a decreased connectivity at the peak of symptoms that later reorganized to normalcy around 6 months. Within these regions of low connectivity existed zones of hyperconnectivity, such changes were notable in the frontal and parietal regions [53].

Evoked potentials are also useful in studying the impact of injury to the CNS, but their utility is primarily in severe TBI with coma. Neurophysiologic studies using TMS and somatosensory testing have shown impaired central neural transmission and increased cortical inhibition in patients with persistent postconcussion symptoms [54]. Sensory and auditory gating tests with somatosensory or auditory evoked potentials with P50 may be helpful to evaluate and provide objective measures for postconcussion syndrome.

While promising, functional imaging modalities, neurophysiological tests, and QEEG are limited to research at specialized centers and not applied to the routine clinical care of patients with mild TBI. Because of lack of standardized protocols and wide variability in results, we do not have enough evidence to support their use at this time. Functional MRI activation changes hold the potential to become a future biomarker for PCS evaluation. Persistence or resolution of PCS is otherwise difficult to determine and is solely dependent on the patient's account.

Biochemical markers: neuron-specific enolase is a commonly used marker for neuronal injury. Neuroprotein termed S-100B is seen in astroglial cells and Schwann cells in the CNS and can enter the CSF, which could become a sensitive marker for brain injury in TBI. Its specificity, however, is poor, and the test is not readily available. Magnetic resonance spectrography can characterize the local levels of N-acetyl aspartate, which is typically reduced along with an increase in local choline levels in TBI patients. Acetylcholine levels have also been shown to be low following TBI [55].

## 7. Treatment of Postconcussion Syndrome

Because of a high variability in onset, duration, and severity of symptomatology, there is a lack of scientifically proven protocols or strategies for treatment of postconcussion syndrome. Recommendations vary based on provider and site expertise. Regardless, certain principles can be applied to treating PCS.

### 7.1. Rest and Sleep

Good quality sleep and rest are paramount in preventing and treating postconcussion syndrome. How long of a rest is needed after a concussion varies. After a head injury, it is a good idea to rest for 48 hours. Rest includes both physical and cognitive rest while cautioning against a long-term rest. Routine nonexertive daily activities are needed to maintain baseline functioning. The duration of rest varies from person to person. It may be unnatural for an active person to cease all activities. In fact, in children and adolescents, early reinitiation of activity and exercises within a week of head injury has shown to help reduce the incidence of postconcussion syndrome [56]. In such scenarios, it would be beneficial to maintain minimal activities of daily life. Aerobic exercises and exercises involving vigorous head or neck movements should be avoided. An athlete with concussion needs to start exercise in 2-3 weeks to avoid physical deconditioning. The most appropriate recommendation would be a graded resumption of activity based on tolerance, starting with 5–10 minutes of cardiovascular exercises in the first week itself. This should take into account the variability caused by age, physical, and psychological factors.

Brain recuperates with sufficient rest and sleep. The quality of sleep and rest is important, as reorganization is facilitated by deeper stages of sleep. Sleep quality is often impaired in patients with postconcussion symptoms. Sleep aids such as melatonin, sleep music, or white noise may be helpful. Medications such as cyclobenzaprine provide dual effects with muscle relaxation and improved sleep. Waking refreshed is a sign of good quality sleep. It may be beneficial for patients with postconcussion syndrome to take afternoon naps. It is very important to set a regular sleep schedule, as it can help steady the deranged circadian rhythms.

### 7.2. Behavioral Measure

Recovery involves a relearning process. Setting a routine for daily activities is the most important behavioral modification to bring the brain back to normalcy. Regular times for waking up and sleep can help put the brain on a schedule. Stimulants such as caffeine should be avoided in the evening. Alcohol and CNS depressants should be avoided at all times. Avoiding social gatherings, social networking, loud noises, and rock music is important. Reducing screen time is necessary whether it be TV, computer screens, or phones. Nature walks at sunrise or sunset in a calm environment is helpful. Gentle instrumental music and light reading from paper or books is encouraged.

### 7.3. Medications

Medications are not the cornerstone of postconcussion therapy. While they provide symptom relief, it can prolong the duration of PCS. Antianxiety medications, antidepressants, and stimulants may be helpful in the acute phase, but their sedative and dependence effects cause slowing of adaptation and clouding of clinical picture. The dose of medication needed at onset may be significantly higher than needed as the patient recovers. Unless the medications effects are identified and tapered early, they can slow recovery and mislead physicians. Worsening premorbid psychiatric conditions is best treated with involvement of a psychiatrist.

Avoidance of cognitive load is important till the brain is ready. A common mistake by patients is to push themselves, hoping for an early recovery. This could become counterproductive. Patients should be advised to stop their tasks and take rest as soon as fatigue sets in. Fatigue is very common in PCS and is an indicator of cognitive or physical limit. If monitored effectively, time to fatigue can be an effective marker for the recovery process. Patients will notice that the time to fatigue increases as their condition improves. Steady improvement in work capacity with a longer “time to fatigue” is an encouraging sign of recovery.

### 7.4. Headaches

The most common cause of these headaches is cervical root irritation. The triggering of the occipital nerves by whiplash often leads to bouts of migraines, dizziness, and neck spasm. Occipital nerve blocks can help break the headache circuit between the cervical roots and spinal nucleus of the trigeminal nerve. Studies using subcutaneous sumatriptan have shown resolution of the trigeminal component of the pain [57]. Once migraine sets in, parenteral migraine medications such as dihydroergotamine (DHE) or sumatriptan can be used to abort the headache. NSAIDs are useful to reduce pain and inflammation associated with muscle strain and spasm. Muscle relaxants at night are helpful for relaxation of neck muscles and improve sleep. It is not advisable to be used in the morning hours, as it may worsen the cognitive difficulties.

Memory foam neck pillows and sleeping on the asymptomatic side are helpful. Chiropractic therapy and supervised neck traction devices are often beneficial. Neck massages and local applicants such as diclofenac cream, herbal balms, and counter irritants also relieve spasms. Low level of daily neck exercise is essential to keep the neck supple.

Acetaminophen and NSAIDs are adequate for mild-to-moderate headaches. Medications such as butalbital/acetaminophen (Fioricet) and opioids should be avoided as it can cause worsening of cognitive issues and create dependence. Propranolol may be useful as a headache prophylactic as well as a regulator of autonomic dysfunction. However, its use is often limited by worsening of depression, sexual dysfunction, and cardiovascular effects. Gabapentin and amitriptyline are generally helpful in reducing nerve sensitivity. Amitriptyline also has antianxiety and antidepressant effects. Both of these medications have the potential for sedation.

### 7.5. Cognitive Rehabilitation

Cognitive rehabilitation begins with a baseline assessment using a panel of tests such as the Montreal Cognitive Assessment Scale (MOCA) or individual tests such as the Trail Making test. Neuropsychiatric testing may be more comprehensive, but often not possible in acute situations. When there are chronic persistent cognitive issues with ADHD-type symptoms, CNS stimulant medications such as methylphenidate can be tried. However, it needs to be avoided where there is potential for addictions, risk for seizures, and in patients with sleep issues. Donepezil has been tried for short-term memory problems related to PCS. Day time sleep issues can be countered with modafinil.

Repeated head impact during the recovery phase is detrimental to the brain's recovery and can cause permanent damage from chronic posttraumatic encephalopathy. It is of utmost importance to avoid further injuries and fall precautions should be discussed with patients.

Cognitive behavioral therapy is useful for mood disorders related to concussion. Breathing and meditation exercises are helpful in improving the autonomic regulation and enhancing attention as an anchor around which the recovery process can happen.

### 7.6. Occupational Therapy

Subjects who have significant cognitive difficulties and work-related issues benefit from occupational therapy. Vision therapy and prism lenses for convergence issues can be considered along with this.

### 7.7. Vestibular and Neck Therapy

The mainstay of therapy in postconcussion syndrome involves the vestibular and neck physical therapy. Vestibular therapy involves exercises to train the vestibular system using oculomotor, head, and trunk exercises aimed at improving balance and vision. This involves tandem standing exercises, Fukuda marching and object tracking, and stabilization exercises. Balance exercises can strengthen the reciprocal balance mechanisms. Visual and oculomotor therapy can help retraining smooth pursuit movements and target fixation. Severe vestibular dysfunction, especially in the acute phase, can be difficult to treat because of triggering of dizziness and nausea and symptoms flare up secondary to neck therapy. Use of vestibular sedatives such as meclizine, benzodiazepines, flunarizine, or betahistine (not available in the US) can be considered in these scenarios. Medications such as gabapentin and amitriptyline can also help reduce the nerve sensitivity, and cyclobenzaprine helps with muscle relaxation, but all of them have a propensity for sedation.

Neck range of movement (ROM) is important for the balance reflexes that prevent falls. Most patients with postconcussion have some components of cervicogenic dizziness and imbalance. Neck therapy should be part of rehabilitation therapy for postconcussion syndrome. Neck physical therapy, chiropractic therapy, and loosening of neck musculature with exercises, massages, warm compresses, and local applicants are helpful.

### 7.8. Novel Therapies

Mindfulness meditation is effective for a variety of individual symptoms that are seen in postconcussion syndrome. Studies with mindfulness meditation have varied results based on the approaches. A randomized trial in patients with moderate TBI and persistent attention deficits did not have significant improvement with short self-directed audio-tape sessions [58], while a pilot study in postconcussion patients and a study in brain injury from trauma and stroke showed significant improvement in cognition, fatigue, and neuropsychological tests [59, 60].

Patients have reported improvement with cannabinoids, whether taken recreationally or medically for other comorbid conditions. Cannabinoids have shown potential for treating individual symptoms associated with PCS [61]. Author has no experience treating PCS patients with cannabinoids.

Hyperbaric oxygen therapy: studies have shown improvement in cognition following hyperbaric oxygen therapy in patients with persistent postconcussion syndrome, even in chronic phases [62]. It is not routinely available or used outside of tertiary care centers.

EEG biofeedback has shown to help reduce postconcussion symptoms in patients who have not responded to conventional therapy [63]. Consumer EEG biofeedback devices are now available, which are affordable, easy, and safe to use. They may help train attention networks, which are deranged in postconcussion syndrome.

Brain stimulation techniques such as transcranial direct current stimulation (tDCS) have shown to boost attention in mild TBI patients when used along with intensive cognitive rehabilitation therapy [64, 65]. Future studies are needed to determine its role.

### 7.9. Reassurance

Last but not the least, reassurance from healthcare providers regarding the temporary nature of this condition is very important. Having clear insight of their deficits is often the cause of worry and frustration. Most patients fear the condition becoming permanent. It has to be emphasized every time that unless repeated concussions occur, recovery is the rule. There needs to be a discussion about various physical and psychological factors causing slow recovery in individual patients. Patients should be advised to focus on their abilities rather than their disabilities and should be encouraged to focus on every successful step they make towards their recovery process.
